# Eating habits and dietary acculturation effects among international college students in the United States

**DOI:** 10.3934/publichealth.2020020

**Published:** 2020-04-27

**Authors:** Amir Alakaam, Amanda Willyard

**Affiliations:** 1 Department of Health and Human Performance, University of Tennessee at Chattanooga, Chattanooga, TN, USA; 2 Department of Education, Health, and Behavior Studies, University of North Dakota, Grand Forks, ND, USA

**Keywords:** dietary supplement, international student, campus food, traditional meal, grounded theory

## Abstract

Although the United States rates as the top host country for international students in the world, the impact of dietary acculturation on their health status is poorly understood. This study used a qualitative approach to gain an in-depth understanding of the factors related to dietary acculturation among international students in U.S. universities. Ten focus groups, of which eight were in two Midwestern U.S. universities and two in a Southern U.S. university (n = 44), were studied. Participants were international, college-aged students enrolled in U.S. universities. Grounded theory method was used for data analysis to develop themes relevant to dietary changes and factors related to dietary acculturation based on the participants' perspectives and experiences. The results showed that most of the participants faced various dietary challenges and health consequences in the United States. Access to food, religious orientation, time constraints, campus environment, and healthcare access were the main factors influencing student diets. A majority of the students reported weight gain due to eating larger portions, unstructured mealtimes, and frequent snacking. Some students reported that they do not eat fruits and vegetables in the United States because they believe that these foods are not fresh, genetically modified, and may contain pesticides. The majority of participants reported taking unprescribed dietary supplements since moving to the United States. Students who transitioned to the typical American diet reported weight gain, increased fatigue, abdominal discomfort, and other health consequences. Universities should seek to develop policies and programs aimed to reduce the impact of acculturation in order to improve the experience of international students in the United States.

## 1. Introduction

The United States hosts more international college students (ICSs) than any other country. According to the Institute of International Education [1], in the academic year of 2016–2017, enrollment reached an all-time high, totaling over one million ICSs in the United States. The top five countries of origin for ICSs were China, India, Saudi Arabia, South Korea, and Canada, accounting for over 55% of all ICSs studying in United States colleges and universities. The diverse cultural backgrounds of ICSs in the United States pose various challenges to be overcome in their host country. Such challenges include learning a new language, adapting to a new culture, handling academic pressures, and potentially making changes to eating habits and food choices [2],[3].

Among the many factors contributing to newcomers adopting the dietary practices of the host country is a process called “dietary acculturation” which defines as a practice of adopting an individual's eating habit and food choices to a new food environment in another community [2]–[5] Researchers have found a relationship between the level of dietary acculturation and risk factors for chronic diseases among different populations [2]–[8]. However, little is known about the dietary acculturation process and its effects on overall health among U.S. ICSs. International students have individual preferences and traditions that may impact their responses to dietary acculturation processes. Additionally, campuses throughout the United States have their own unique environments that may impact the food choices of ICSs [2],[9].

Published data assessing the dietary acculturation process in U.S. campuses is scarce. Because the U.S. is the top host country for ICSs, such data are necessary to assist colleges and universities in developing programs aimed at mitigating the impact of dietary acculturation and improving the ICS experience in the United States [2],[10]. The purpose of this study was to examine the eating habits and changes in food choices, as well as to gain an in-depth understanding of the factors related to dietary acculturation effects among the ICS population in the U.S. The current study is part of a larger research project initiated in 2013 that has been conducted at several universities in the Northern, Southern, and Midwestern areas in the United States [2],[11]. This project aimed to obtain preliminary data for comparison and analysis with previous and future quantitative findings to bridge the gap in the existing literature.

## 2. Materials and methods

### 2.1. Study design

The study used a focus group qualitative research method for its design. Grounded theory was used for collecting and analyzing the data to effectively gain insights into the dietary changes and factors related to dietary acculturation among the ICSs. The grounded theory is a qualitative approach enabling themes and findings to emerge from the data collected, and serves as a broad method following a systematic, yet flexible, process to examine data, create codes, and generate concepts that emerge from participants' opinions and experiences [12]–[14].

The study received approval from the University of Southern Mississippi and the University of North Dakota Institutional Review Boards.

### 2.2. Participants

The research involved a wide range of participants from three different universities. Two universities are located in the Midwestern U.S. region, and one university is located in the Southern U.S. region. All the universities are public state universities and located in U.S. metropolitan areas.

The researcher recruited the initial participants by contacting international and study abroad centers to advertise the research. Snowball sampling was used to recruit further participants. Participants were eligible to participate if they were international students, currently enrolled in U.S. universities, lived in the United States for three months to five years, were fluent in the English language, and were between the ages of 18 and 40 years. The inclusion criteria for international students were based on the U.S. Citizenship and Immigration Services (USCIS) category of student visa [15]. Students were considered international if they entered the United States as a holder of one of the following visas: F-1 Academic student visa, J-1 Exchange visitor visa, and M-1 Vocational student visa [15].

Participants were compensated for volunteering their time by being gifted a meal in a restaurant near the meeting location. Informed consent was obtained from the participants prior to the interview.

### 2.3. Data collection and analysis

Data were collected using a semi-structured format interview moderated by the researcher. All participants were asked a set of core open-ended questions based on the interview guide, for example, participants were asked the following: Tell me about what you ate before coming to the United States? How has your diet changed since coming to the United States? How do you decide on what food you eat in the United States? Additionally, participants completed a demographic questionnaire. The interviews were audiotaped and subsequently transcribed verbatim.

Two researchers, trained in analyzing qualitative data, performed systematic coding of themes and developed subsequent categories based on the grounded theory method [12]–[14]. The concepts were labeled and organized into categories consistent with open-coding procedures. Data were analyzed to gain an understanding of participants' thoughts and opinions. Axial coding was performed after open coding, which enabled the researchers to explore how each of the categories related to one another, which resulted in the transformation of categories into subcategories and identification of broader thematic modules. The final process was selective coding, which allowed the researchers to identify conceptual ideas that integrated the existing data. The analysis steps were not strictly sequential; rather, the process consisted of moving forward and backward to constantly re-examine data and refine the overall model. Study procedures and measures were also reviewed by a qualified external researcher to ensure the trustworthiness of the qualitative method.

## 3. Results

The study included ten focus groups (n = 44): eight groups (32 participants) in two Midwestern U.S. universities and two groups (12 participants) in a Southern U.S. university. Each focus group comprised 3–6 participants, and lasted around 60–90 minutes. The mean age of participants was 23.69 years (range 18–32 years); about 55% of participants were male and 45% were female, about 46% of participants were Asian, and 32% were White. Table 1 presents participants' characteristics.

**Table 1.** Characteristics of the sample (n = 44).

Characteristic	n (%)
Region of Origin	
Asia	20 (45.5)
Middle East	10 (22.7)
Africa	4 (9.1)
North America	3 (6.8)
South America	3 (6.8)
Europe	3 (6.8)
Australia (Oceania)	1 (2.3)
Race	
Asian	20 (45.5)
White	14 (31.8)
African American	6 (13.6)
Hispanic	4 (9.1)
Religious orientation	
Christian	15 (34.1)
Muslim	12 (27.3)
No Religion	11 (25.0)
Hindu	5 (11.4)
Buddhist	1 (2.3)
Current Educational Level	
Undergraduate	34 (77.3)
Graduate	10 (22.7)
Current Region of Residence	
Midwestern United States	32 (72.7)
Southern United States	12 (27.3)
Time lived in the United States	
2–5 years	24 (54.5)
6 months–2 years	20 (45.5)
Current Place of Residence	
Off-Campus	21 (47.7)
On-Campus	17 (38.6)
No answer	6 (13.6)
Relationship Status	
Single	40 (90.9)
Married	4 (9.1)
Employment Status	
Unemployed	32 (72.7)
Part-time	10 (22.7)
Full-time	2 (4.5)

Data analysis generated five themes: (1) eating habits in the home country; (2) eating habits in the United States; (3) factors influencing eating habits in the United States; (4) health consequences of these changes, and (5) Dietary supplement intake. See Table 2 for main findings and selected quotes from participants.

**Table 2.** Overview of themes with representative quotes from participants.

Themes	Main Findings	Participants' Quotes
Eating Habits in the Home Country	Meals include fresh ingredients, rice, noodles, beans, or meat. The mother, or housekeeper, typically prepares meals. Meals are structured and eaten at home. It is uncommon to eat dinner late. Breakfast is optional. Tea, milk, and water are the main beverages.	“Lunch is mainly rice and bread with some meat, and veggies.” “My meals depended on my mother, whatever she wants to make at that time.” “Tea is what we have mainly in India, the day starts with it, or milk.”
Eating Habits in the United States	Meal is large in size and high in preservatives, sugars, and fats. Adaptation to American foods, including pizza, fries, and sandwiches. Eating buffet-style at the dining center. Diet includes late-night meals. Breakfast is the least important meal. Soda is the main beverage consumed.	“I ate a lot of pizza, burgers, and cheese steaks.” “American portion sizes fill up the entire plate, you get like two or three times the size we get back home at a restaurant. I am not kidding, it's huge, like crazy huge.” “The large drinks in Saudi Arabia, here it's considered as small”
Health Consequences from Changes in Eating Habits	Weight gain from adopting an American diet, unstructured mealtimes, and stress. Weight loss from resisting an American diet. Weight loss, or gain, due to different weight perceptions. Acid reflux, ulcers, acne, and increased fatigue. Taking unprescribed dietary supplements.	“In Korea, I used to eat healthy food, but when I came to America, I always eat hamburgers, pizza, and pasta, so I gained weight. And it's fat.” “I got a lot of stomach problems since I came here because I eat fast food and drink three or four pops a day.” “When I first came here, I gained a massive amount of weight.” “It's very cold and not exposed to the sun that much. I never talked to my doctor actually, about that. My friends said you should take Vitamin D and supplement.” “I've gained a lot of weight in the first semester, it's the sweets and the cakes that really got me, just surrounded by all those options of sweets.”

### 3.1. Eating habits in the home country

Participants ate traditional meals that were culturally familiar to them. The meals consisted of rice, noodles, chicken, pork, soup, beans, or eggs. The majority of participants ate non-vegetarian diets. Beverages typically included tea, milk, juice, or water.

The mother or a housekeeper typically prepared meals; it was uncommon for other members of the family to be able to cook. Meals were typically structured around three courses, namely breakfast, lunch, and dinner. Although mealtimes varied across the sample, it was uncommon to eat dinner late at night.

### 3.2. Eating habits in the United States

Participants typically first lived in a dorm after they moved to the United States. Most of the participants indicated that when, living in a dorm, they were required to buy a meal plan for the university's dining centers. The dining centers are all-you-can-eat buffet style, which was a new experience for the majority of the participants. The participants referred to the food available at the dining centers as “American foods” or “American diet”.

The participants mentioned that the American diet has larger portion sizes, and tends to have larger quantities of preservatives, sugars, fats, and processed foods, including genetically modified foods. Many participants explored or adapted the American foods into their diet. Participants reported their meal choices as pizza, pasta, sandwiches, chicken, beef, French fries, salad, and desserts at the dining center. A large percentage of participants that reported having a vegetarian diet in their home country switched to a non-vegetarian diet as an easy way to ensure that their diet in the United States comprised adequate protein.

Participants additionally chose soda as their beverage of choice. Some students learned how to cook, returned to smaller portion sizes, and gave up drinking soda during their first year of studies in the university. Participants who skipped breakfast in their home countries also skipped breakfast in the United States. Breakfast was notably the least important meal of the day among the participants.

Meals were typically unstructured and dependent on class schedules. Participants also started including late-night meals and snacks into their diet owing to classes at night, late-night studying, or meal availability at their dining centers. Students who lived more than two years in the United States were better able to adapt to the American diet and reported more changes to their home country eating habits compared with students who lived less than two years.

### 3.3. Factors influencing eating habits in the United States

Factors that influence eating habits and food choices of ICSs vary based on the participants' perspectives and experiences. Data analysis identified six factors that may explain dietary changes. among the participants.

#### 3.3.1. Food access

Participants found it easier and more convenient to obtain American food than traditional food. Students who adopted an American diet did so out of convenience and easier food access, not just to save money. The main factors that resulted in difficulties obtaining access to traditional foods were transportation and food availability. Participants from the Southern university indicated a lack of public transportation in general. Participants from the Midwestern universities indicated that buses (public transportation) were only available once an hour, and it was unpleasant to walk to the bus stop and wait there in the winter.

The Midwestern ICSs reported that bus stops were not close enough to the international markets to be able to walk to them in the cold weather. Students also reported that taxis were inconvenient because they took up to 30 minutes to pick them up. Students who did not own a car reported going grocery shopping on average 1–2 times a month. Students who did own a car reported grocery shopping more frequently, averaging once a week. International market availability was also an important factor influencing ICSs food access. Participants who lived in urban cities in the Midwestern U.S. reported better access to traditional stores than those who lived in Southern U.S. Some participants reported that the launch of convenient ridesharing services, such as Uber, positively impacts their access to traditional foods.

#### 3.3.2. Personal food preference

The majority of students who preferred traditional foods to American foods moved out of the dorms and into an apartment in order to be able to cook for themselves. The students who preferred fast food and convenience were better able to adapt to the American diet. The majority of the students mentioned that they were unable to buy produce at U.S. grocery stores that was as fresh as at the stores in their home countries. Some students reported that they do not eat fruits and vegetables in the United States because they believe that these foods are not fresh, genetically modified, and may contain pesticides. However, a few students from the Midwestern universities mentioned that they had visited farmers markets and were pleased with the quality of produce available there, and consumed more fruits and vegetables as a result.

#### 3.3.3. Religion and culture

Students with strong family and personal values that impacted their diets, such as those who only ate vegetarian or halal food, found it difficult to meet their obligations due to limited access to such foods in their residences and on campus.

#### 3.3.4. Time constraints

A majority of students reported that their classes and coursework load made it difficult to spend time planning meals and cooking. Students who did not want to make cooking a priority preferred food that were convenient and easily accessible.

#### 3.3.5. Campus environment

Students reported that the campus environment plays a major role in their eating habits. Students living in dorms were introduced to a wide variety of American foods at the dining centers that had never been a part of their diets before moving to the United States. Although the dining centers offer healthy options, lack of self-control made it difficult to make healthy choices when surrounded by a variety of unhealthy options.

#### 3.3.6. Healthcare access

Students indicated that U.S. healthcare services are expensive, and, as a result, they seek care only in emergencies. Participants from the Southern university mentioned either having limited healthcare coverage or that the high cost of health insurance was a contributor to their food choices. Students mentioned that they did not want to be unwell or have unhealthy eating habits because their health insurance would not cover their health expenses.

Table 3 provides an overview of the factors influencing eating habits in the United States, with quotes from participants.

**Table 3.** Factors influencing students' eating habits in the united states, with quotes from participants.

Factors	Main Finding	Participants' Quotes
Food Access	It is easy and convenient to obtain American food. Shopping less frequently owing to limited transportation. Foods are more expensive than in the home country. International stores are the main source of food.	“The international store that we have has been a great help, they are the main source for most of the internationals.” “I feel like here, if you want to eat healthy actually it's costly.” “My choices are very limited in the U.S. For example, a cheeseburger here has a beef in it. But in India, it's still a vegetarian patty.” “I was living on campus and I wanted fruit, I had to ask for a ride. It is always been hard.”
Personal Food Preference	Concerns that produce are genetically modified and contain pesticides. Satisfaction with produce available at the farmers' market produce, which increases fruit and vegetable intake. Unable to buy produce from grocery stores that is as fresh as that available in their home countries.	“I'm from Saudi Arabia, I struggle with the meat, lamb. It's not easy to find and if you find it, it's really expensive” “We eat different types of vegetables back home like okra. Here you cannot find it easily and it's not fresh.” “I was eating cheese pizza for a few weeks, then, I was sick of it. I tried making traditional meal, but everything tasted different.”
Religious and Culture	Students following specific religions and family values found it difficult to neglect their obligations. Unable to purchase foods related to specific religions in American stores Limited vegetarian food options available on campus	“I'm a vegetarian, in India, I didn't eat eggs. But since I moved to U.S., I started eating eggs. That was a major change for me.” “I'm a Muslim and I have to eat certain kind of food. I asked around about halal meat. We call it halal just like Kosher. It's not available here. So, I was like, I'll just be vegetarian.”
Time Constraints	Coursework load makes it difficult to spend time planning meals and cooking. Students prefer food choices that are convenient and easily accessible.	“Right now, all I really care about is just my school. I don't have time to eat healthy.” “You have your own family values that you want to respect, but with the cost and time, it is all just too much of an obligation.”
Campus Environment	Self-control was difficult owing to the wide variety of foods on campus. Many students had never experienced eating buffet-style before eating on campus.	“Even in the dining center we've got so many choices of sweet. It's very hard to stay away from it.” “It is not easy to find healthy food options on campus.”
Healthcare Access	U.S. healthcare service is expensive. Limited coverage or high cost of health insurance in the United States.	“Here in U.S., I'm more conscious because the healthcare service is very expensive and I don't want to have any health problem in my body and go to hospital and spend money.”

### 3.4. Health consequences of dietary changes

Participants self-reported various health consequences in the United States due to dietary changes. A majority of the students reported gaining weight because of stress, eating larger portions and unhealthy items at the dining center, unstructured mealtimes, and frequent snacking. Weight perception was also a factor for students who attempted to gain weight on purpose after arriving in the United States; for example, participants reported that males in the United States have a more athletic and stocky build compared with males in their home country.

Some students reported losing weight after moving out of their dorms, and mentioned that weight gain played a major role in their decision to leave their dorms and move into a residence with a kitchen. Few students mentioned that they purposely skipped meals and exercised to lose weight faster.

Muslim students reported weight loss due to not consuming sufficient amounts of food, because many food items available at their dining centers contained pork. Some vegetarian students reported weight loss as a result of the lack of vegetarian options besides salads. Participants also reported constantly feeling hungry. Other health consequences reported by ICSs included acid reflux, ulcers, acne, and increased fatigue.

### 3.5. Dietary supplement intake

Several students reported that they regularly take dietary supplements as a potential solution to the health consequences of their dietary changes. Most of the students consumed dietary supplements if they did not feel they were getting adequate nutrition, or if they experienced fatigue. The majority of students started taking dietary supplements without a prescription from a healthcare provider, and did not start taking them until they moved to the United States. Students reported that their friends or family would typically recommend taking a Vitamin D supplement especially during the winter. The most common dietary supplements students reported taking were multivitamins, protein powder, vitamin D, probiotics, iron, vitamin C, vitamin B12, calcium, omega 3, and weight-loss supplements.

## 4. Discussion

This study explores the impact of dietary acculturation processes among ICSs in the Midwestern and Southern U.S. One of the main challenges faced by many first-year ICSs was living in dormitories; among the majority of the ICSs, eating habits changed because of the meal plans that are required when students live in dorms. Previous studies have mentioned that dining services on campus are a major contributing factor to the decision of ICSs to move off-campus and prepare food at home [2],[10].

One of the other factors that has been frequently found to be a key contributor to the dietary acculturation process in ICSs is transportation [2],[10],[16]. A majority of our participants reported shopping for groceries less often towing to limited transportation. Car-pooling was found to be the preferred mode of transportation, especially in the Midwestern universities, because of cold weather and public transportation schedules. While we were conducting our study at one of the Midwestern universities, our participants informed us that Uber (a convenient and inexpensive worldwide ridesharing service that can be requested via a mobile app) had launched in the city [17]. We learned from these participants that Uber had a positive impact on their frequency of grocery shopping. It would be informative to determine whether a convenient transportation service such as Uber can make a significant positive impact on the ICSs food access and dietary changes. To our knowledge, no previous studies have examined the implications of Uber or similar services as part of their research data in relation to transportation.

In the present study, we posed questions related to dietary supplements that have not been included in our previous research on ICSs populations [2]. Our participants reported consuming a variety of dietary supplements based on recommendations by friends, without consulting healthcare providers. Dietary supplements are widely available and their intake has important consequences, especially among college-age students [18],[19]. The consequence of dietary supplement consumption needs to be further tested among ICSs in future research using a quantitative research design and specific questionnaires that address dietary supplement intake.

U.S. policies and federal regulations present challenges to conducting research on ICSs in the United States. International students have a number of restrictions regarding their immigration status that limit their ability to undertake employment and earn an income in the United States [15]. It has been argued that paying ICSs incentives (e.g. money or a gift card) in exchange for their participation in research constitutes employment. As a result, many U.S. institutions prohibit ICSs from receiving gifts of money (including store and restaurant gift cards) as compensation to avoid the possibility of violating federal regulations, leading to complicated paperwork [20]. Indeed, this is what happened in our study. Our institute did not allow us to provide ICSs participants with any type of monetary compensation, whether it was a gift card or cash. We believe that research and interventions targeting ICSs may be limited because of the difficulties in recruiting ICSs as participants due to these restrictions. Compensation for time and effort is an important motivator for many individuals to take part in research, regardless of their financial status [21],[22].

Although this study has a number of strengths, there are also limitations. While ICSs from the two geographical areas in the United States focused on in this study may share similar experiences with other ICS populations, caution should be exercised when attempting to generalize the present findings. The analytical style in this qualitative study used subjective interpretation to identify themes; however, three independent coders were used to check for consistency and to reach trustworthiness in the analysis.

## 5. Conclusions

This qualtative study explored eating habit and food choices changes, and factors related to dietary acculturation in the ICS population, specifically, international students enrolled in universities in the midwestern and northern regions of the United States. Future research should aim to examine the effects of dietary acculturation and its long-term consequences on health, mental wellbeing, and performance among a larger sample of ICSs from U.S. higher education institutes. The application of quantitative method with 24-hour food records, assisted by extensive health and dietary analysis, may represent a promising direction for future research.

The ultimate goal of this research is to provide universities an evidence-based program in order to maximize ICSs adaptation to U.S. culture, and to minimize the challenges they experience during dietary acculturation. This may include provision of adequate on-campus educational nutrition programs to eliminate barriers to future ICSs who lack knowledge of the food environment in their host country.
